# Statistical design considerations for pilot studies transitioning therapies from the bench to the bedside

**DOI:** 10.1186/1479-5876-2-37

**Published:** 2004-10-28

**Authors:** Rickey E Carter, Robert F Woolson

**Affiliations:** 1 Department of Biostatistics, Bioinformatics and Epidemiology, 135 Cannon Street, Suite 303, Medical University of South Carolina, Charleston, SC 29425, USA

## Abstract

Pilot studies are often used to transition therapies developed using animal models to a clinical setting. Frequently, the focus of such trials is on estimating the safety in terms of the occurrence of certain adverse events. With relatively small sample sizes, the probability of observing even relatively common events is low; however, inference on the true underlying event rate is still necessary even when no events of interest are observed. The exact upper limit to the event rate is derived and illustrated graphically. In addition, the simple algebraic expression for the confidence bound is seen to be useful in the context of planning studies.

## Introduction

In the translational research setting, statisticians often assist in the planning and analysis of pilot studies. While pilot studies may vary in the fundamental objectives, many are designed to explore the safety profile of a drug or a procedure [1,2]. Often before applying a new therapy to large groups of patients, a small, non-comparative study is used to estimate the safety profile of the therapy using relatively few patients. This type of investigation is typically encountered in the authors' experiences as collaborating biostatisticians at our General Clinical Research Center as well as developing applications addressing the National Institutes on Health Roadmap Initiative .

In the context of pilot studies, traditional levels of *α *(the Type I error rate) and *β *(the Type II error rate) may be inappropriate since the objective of the research is not to provide definitive support for one treatment over another [3]. For example, the null hypothesis in a single arm pilot study might be that the tested intervention produces a safety profile equal to a known standard therapy. A Type I error (rejecting the null hypothesis when it is false) in the context of this preliminary investigation would encourage additional examination of the treatment in a new clinical trial. This is in contrast to a Type I error in a Phase III/IV clinical trial in which the error could result in widespread exposure of an ineffective treatment. Allowing for a less stringent Type I error rate is critical when trying to transition therapies from the animal models to clinical practice since it identifies a greater pool of potential therapies that could undergo additional research in humans.

Similarly, power (1 - *β*) is of less practical importance in a single arm, non-comparative (or historically controlled) pilot study since the results would almost always require confirmation in a controlled trial setting. Shih *et al *[4] extend the deviations from traditional hypothesis-driven analyses to suggest preliminary investigations should focus on observing responses at the subject level rather than testing a treatment's estimated mean response. In the section that follows, we will relate these notions under the context of safety data analysis and provide interpretations that can be used for sample size considerations.

## Methods

For ease of presentation, assume the pilot study will involve *n *independent patients for which the probability of the adverse event of interest is *π*, where 0 <*π *< 1. A 100 × (1 - *α*)% confidence interval is to be generated for *π *and an estimate of the sample size, *n*, is desired. Denote *X *as the number of patients sampled who experience the adverse event of interest. Then, the probability of observing *x *events in *n *subjects follows the usual binomial distribution. Namely,



Denote *π*_*u *_as the upper limit of the exact one-sided 100 × (1 - *α*)% confidence interval for the unknown proportion, *π *[5]. Then *π*_*u *_is the value such that



A special case of the binomial distribution occurs when zero events of interest are observed. In pilot studies with relatively few patients, this is of practical concern and warrants particular attention. When zero events are realized (i.e., *x *= 0), equation (1) reduces to

(1 - *π*_*u*_)^*n *^= *α*.

Accordingly, the upper limit of a one-sided 100 × (1 - *α*)% confidence interval for *π *is

*π*_*u *_= 1 - *α*^1/*n*^.     (2)

The resulting 100 × (1 - *α*)% one-sided confidence interval is (0, 1 - *α*^1/*n*^).

**Figure 1:**
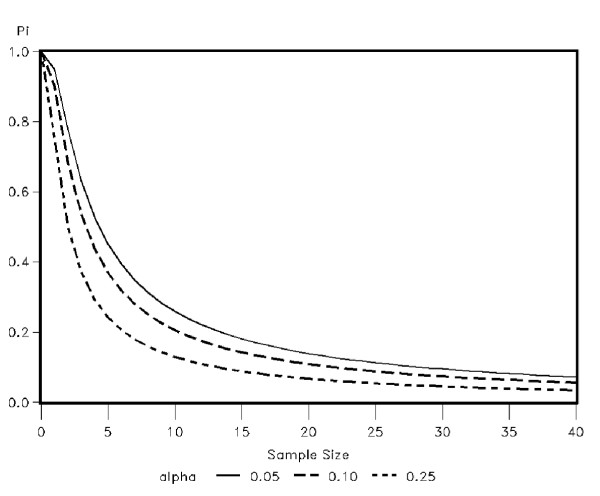
Upper limit of the 100 × (1 - *α*)% one-sided confidence interval for the true underlying adverse event rate, *π*, for increasing sample sizes when zero events of interests are observed

Graphically, one can represent this interval on a plot of *π *against *n *as illustrated in Figure 1 for *α *= 0.05, 0.10 and 0.25. As the figure illustrates, for relatively small sample sizes, there is a large amount of uncertainty in the true value of *π*. It is critical to convey this uncertainty in the findings and to guard against inferring a potential treatment is harmless when no adverse effects of interest are observed with limited data. Louis [6] also cautioned the clinical observation of zero false negatives in the context of diagnostic testing stating that zero false negatives may generate unreasonable optimism regarding the rate, particularly for smaller sample sizes.

Furthermore, one can consider using (2) in other clinically important manners. For instance, an investigator may be planning a pilot study and want to know how large it would need to be to infer with 100 × (1 - *α*)% confidence that the true rate did not exceed a pre-specified *π*, say *π*_0_, given that zero adverse events were observed. Using (2), it follows that:



To illustrate the utility of this solution, consider the following example. Ototoxicity is well documented with increasing doses of cisplatin, a platinum-containing antitumoral drug that is known to be effective against a variety of solid tumors. It is of clinical interest to identify augmentative therapies that can alleviate some of the cell death since up to 31% of patients receiving initial doses of 50 mg/m^2 ^cisplatin are expected to have irreversible hearing loss [7,8]. Therefore, it is desirable to rule out potential treatments not consistent with this rate of hearing loss before considering more conclusive testing. Using equation (3), we would conclude that the augmentative therapy has a hearing loss rate less than 0.31, at the 90% confidence level, if a total of 7 patients are recruited and all 7 do not experience ototoxicity. Therefore, an initial sample size of 7 patients would be sufficient to identify augmentative therapies, such as heat shock or antioxidant supplements, that demonstrate preliminary efficacy in humans. In the event one or more ototoxic events are observed, then the results in relationship to the historical rate (31% in this example) may not be statistically different. The results of several of these pilot studies could then be used to rank-order potential therapies thereby proving an empirically justified approach to therapy development.

## Conclusions

In translational research, it is common to explore the adverse event profile of a new regimen. In this note, we illustrate how a simple expression has utility for the generation of confidence intervals when zero events are observed. A more comprehensive and methodological treatment of inference with zero events can be found in Carter and Woolson [9], and Winkler *et al *[10], which treats the issue from a Bayesian statistical viewpoint. This commentary and related works have implications as a practical finding for the interpretation of clinical trial safety data and offer clinicians advice on the range of adverse event rates that can be thought to be consistent with the observation of zero events. The presented formula offers more flexibility than the "rule of 3" approximation [11] since it allows for the specification of significance levels other than *α *= 0.05. The ability to choose the significance level might be important when designing or interpreting preliminary data obtained from a pilot study. In summary, small sample sizes and a focus on safety are often associated with translational research, and the statistical approaches to these studies may need to deviate from traditional, hypothesis-driven designs.

## Competing interests

The author(s) declare that they have no competing interests.

## Authors' Contributions

RC and RW contributed to the conceptualization, writing and editing of this manuscript.

## References

[B1] Spilker B (1991). Guide to Clinical Trials.

[B2] Friedman LM, Furberg C, DeMets DL (1998). Fundamentals of Clinical Trials.

[B3] Schoenfeld D (1980). Statistical considerations for pilot studies. International Journal of Radiation Oncology, Biology, Physics.

[B4] Shih WJ, Ohman-Strickland PA, Lin Y (2004). Analysis of pilot and early phase studies with small sample sizes. Statistics in Medicine.

[B5] Clopper CJ, Pearson ES (1934). The use of confidence or fiducial limits illustrated in the case of the binomial. Biometrika.

[B6] Louis TA (1981). Confidence intervals for a binomial parameter after observing no successes. The American Statistician.

[B7] Grau J, Estape J, Cuchi M, Firvida J, Blanch J, Ascaso C (1996). Calcium supplementation and ototoxicity in patients receiving cisplatin. Br J Clin Pharmacol.

[B8] Kovach J, Moertel C, Schutt A, Reitemeier R, Hahn R (1973). Phase II study of cis-diamminedichloroplatinum (NSC-119875) in advanced carcinoma of the large bowel. Cancer Chemother Rep.

[B9] Carter RE, Woolson RF Safety Assessment in Pilot Studies When Zero Events Are Observed. Proceedings of International Conference On Statistics in Health Sciences.

[B10] Winkler RL, Smith JE, Fryback DG (2002). The Role of Informative Priors in Zero-numerator Problems: Being Conservative Versus Being Candid. The American Statistician.

[B11] Lewis JA (1981). Post-marketing surveillance – how many patients?. Trends in Pharmacological Sciences.

